# Crystal structure of 3-(adamantan-1-yl)-4-(4-chloro­phen­yl)-1*H*-1,2,4-triazole-5(4*H*)-thione

**DOI:** 10.1107/S2056989015000596

**Published:** 2015-01-17

**Authors:** Reem I. Al-Wabli, Ali A. El-Emam, Obaid S. Alroqi, C. S. Chidan Kumar, Hoong-Kun Fun

**Affiliations:** a Department of Pharmaceutical Chemistry, College of Pharmacy, King Saud University, PO Box 2457, Riaydh 11451, Saudi Arabia; b King Abdullah Institute for Nanotechnology (KAIN), King Saud University, Riyadh 11451, Saudi Arabia; c X-ray Crystallography Unit, School of Physics, Universiti Sains Malaysia, 11800 USM, Penang, Malaysia; d Department of Chemistry, Alva’s Institute of Engineering & Technology, Mijar, Moodbidri 574225, Karnataka, India

**Keywords:** crystal structure, adamantane, 1,2,4-triazole, starting material, hydrogen bonding

## Abstract

The title compound, C_18_H_20_ClN_3_S, is a functionalized triazoline-3-thione derivative. The benzene ring is almost perpendic­ular to the planar 1,2,4-triazole ring [maximum deviation = 0.007 (1) Å] with a dihedral angle of 89.61 (5)° between them and there is an adamantane substituent at the 3-position of the triazole­thione ring. In the crystal, N—H⋯S hydrogen-bonding inter­actions link the mol­ecules into chains extending along the *c*-axis direction. The crystal packing is further stabilized by weak C—H⋯π inter­actions that link adjacent chains into a two-dimensional structure in the *bc* plane. The crystal studied was an inversion twin with a 0.50 (3):0.50 (3) domain ratio.

## Related literature   

For the biological activity of adamantane derivatives, see: Lorenzo *et al.* (2008 ▸); Wang *et al.* (2013 ▸); Kadi *et al.* (2010 ▸); Balzarini *et al.* (2009 ▸); Protopopova *et al.* (2005 ▸); Vernier *et al.* (1969 ▸). For the biological activity of adamantyl-1,2,4-triazole derivatives, see: El-Emam & Ibrahim (1991 ▸); Al-Abdullah *et al.* (2014 ▸); El-Emam *et al.* (2004 ▸, 2013 ▸). For related adamantyl-1,2,4-triazole structures, see: El-Emam *et al.* (2012 ▸), Al-Tamimi *et al.* (2013 ▸), Al-Omary *et al.* (2014 ▸); Almutairi *et al.* (2012 ▸). For the synthesis of the title compound, see: Al-Deeb *et al.* (2006 ▸).
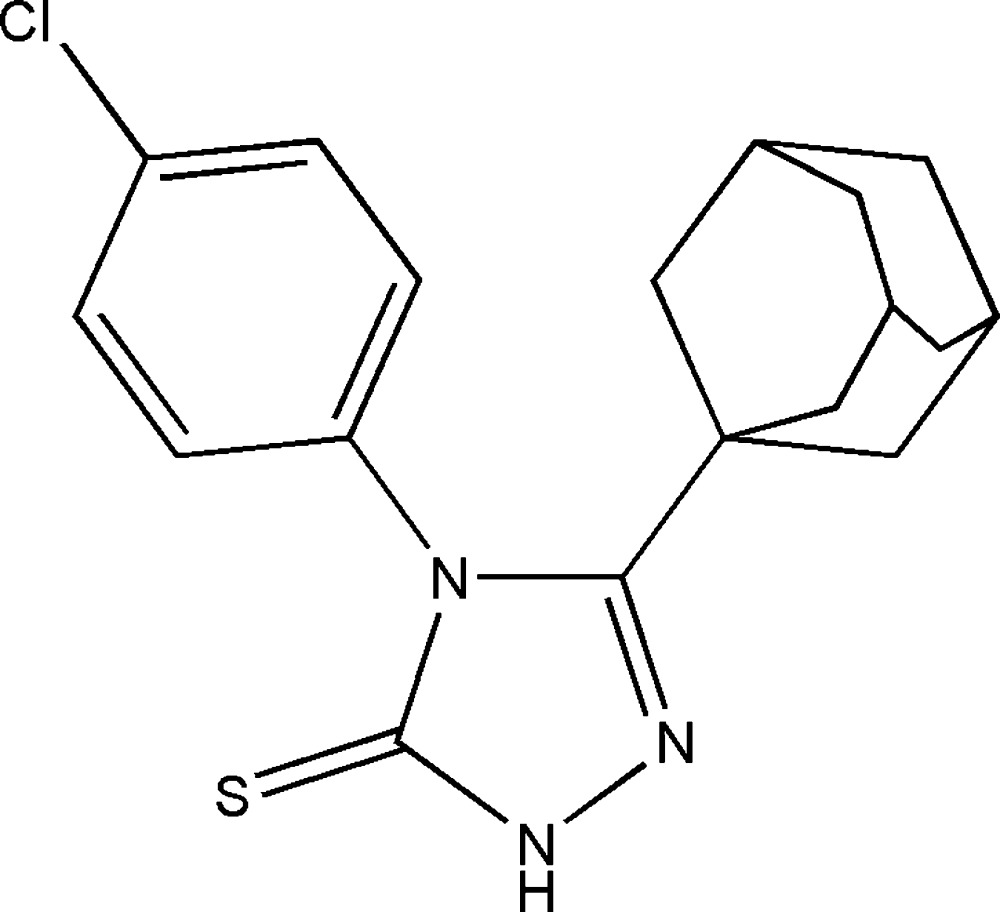


## Experimental   

### Crystal data  

C_18_H_20_ClN_3_S
*M* *_r_* = 345.88
Tetragonal, 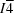

*a* = 23.1302 (5) Å
*c* = 6.4100 (2) Å
*V* = 3429.39 (18) Å^3^
*Z* = 8
Mo *K*α radiation
μ = 0.35 mm^−1^
*T* = 150 K
0.68 × 0.29 × 0.26 mm

### Data collection  

Bruker APEXII CCD diffractometer
106262 measured reflections
11408 independent reflections
10584 reflections with *I* > 2σ(*I*)
*R*_int_ = 0.033

### Refinement  

*R*[*F* ^2^ > 2σ(*F* ^2^)] = 0.030
*wR*(*F* ^2^) = 0.082
*S* = 1.06
11408 reflections
213 parameters
H atoms treated by a mixture of independent and constrained refinement
Δρ_max_ = 0.53 e Å^−3^
Δρ_min_ = −0.31 e Å^−3^
Absolute structure: Flack (1983 ▸), 5353 Friedel pairs
Absolute structure parameter: 0.50 (3)

### 

Data collection: *APEX2* (Bruker, 2009 ▸); cell refinement: *SAINT* (Bruker, 2009 ▸); data reduction: *SAINT*; program(s) used to solve structure: *SHELXS97* (Sheldrick, 2008 ▸); program(s) used to refine structure: *SHELXL2013* (Sheldrick, 2015 ▸); molecular graphics: *SHELXTL* (Sheldrick, 2008 ▸); software used to prepare material for publication: *SHELXTL* and *PLATON* (Spek, 2009 ▸).

## Supplementary Material

Crystal structure: contains datablock(s) I, global. DOI: 10.1107/S2056989015000596/sj5439sup1.cif

Structure factors: contains datablock(s) I. DOI: 10.1107/S2056989015000596/sj5439Isup2.hkl

Supporting information file. DOI: 10.1107/S2056989015000596/sj5439Isup3.cml

**Fig. 1.**
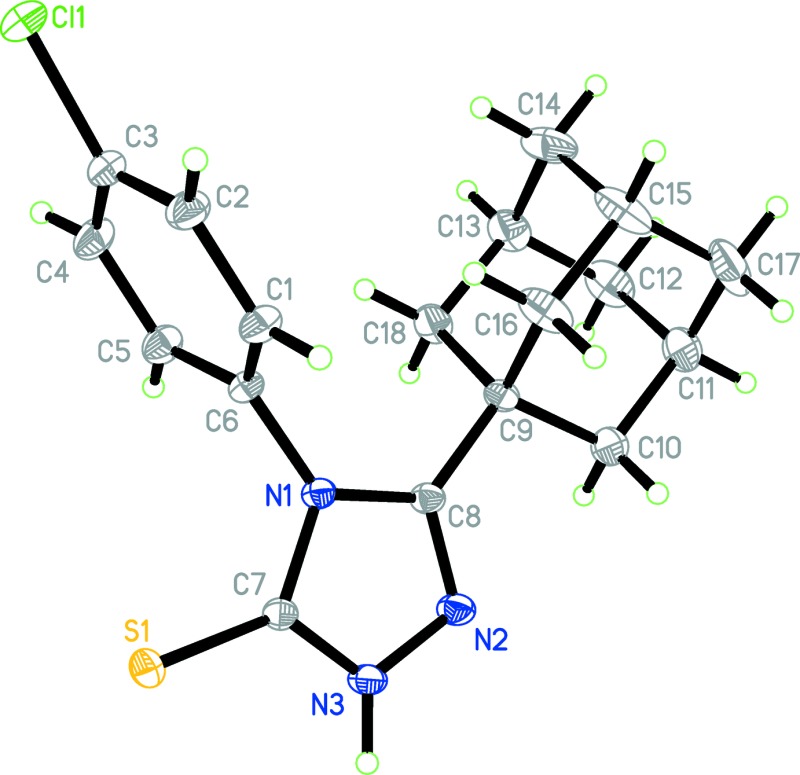
The molecular structure of the title compound with atom labels and 50% probability displacement ellipsoids.

**Fig. 2.**
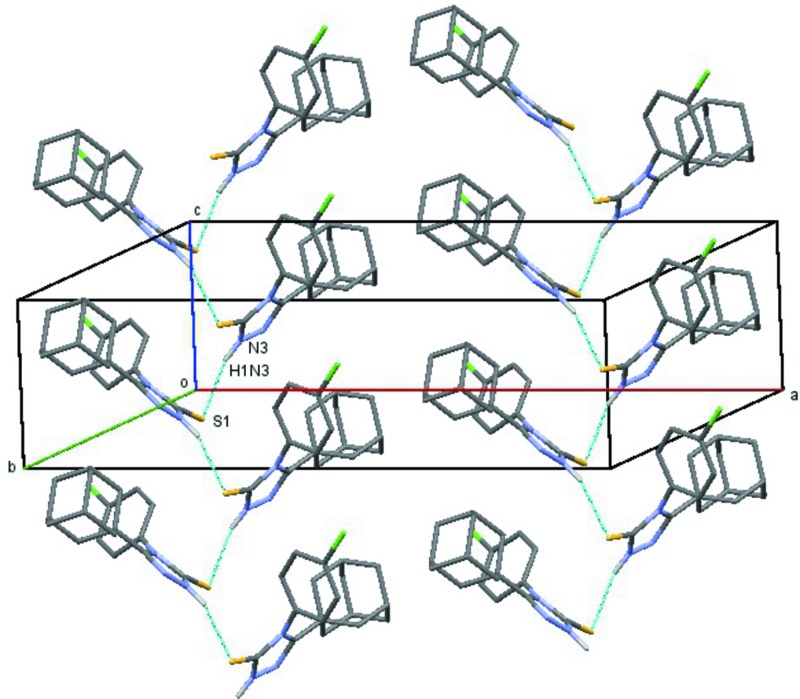
Crystal packing of the title compound, showing the N–H···S hydrogen bonding interactions (Table 1) as dashed lines linking the molecules into chains extending along the c axis direction. Other H-atoms are omited for clarity.

. DOI: 10.1107/S2056989015000596/sj5439fig1.tif
The mol­ecular structure of the title compound with atom labels and 50% probability displacement ellipsoids.

c . DOI: 10.1107/S2056989015000596/sj5439fig2.tif
Crystal packing of the title compound, showing the N–H⋯S hydrogen bonding inter­actions (Table 1) as dashed lines linking the mol­ecules into chains extending along the *c* axis direction. Other H-atoms are omited for clarity.

CCDC reference: 1042916

Additional supporting information:  crystallographic information; 3D view; checkCIF report

## Figures and Tables

**Table 1:** Hydrogen-bond geometry (, ) *Cg*2 is the centroid of the C1C6 phenyl ring.

*D*H*A*	*D*H	H*A*	*D* *A*	*D*H*A*
N3H1*N*3S1^i^	0.92(2)	2.46(2)	3.3253(9)	158.4(19)
C13H13*A* *Cg*2^ii^	0.98	2.97	3.8881(13)	156

Symmetry codes: (i) 
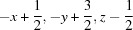
; (ii) 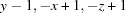.

## supplementary crystallographic information

### S1. Chemical context

Adamantane derivatives have long been known for their diverse biological activities (Wang *et al.*, 2013) including anti­viral activity against influenza (Vernier *et al.*, 1969) and HIV viruses (El-Emam *et al.*, 2004; Balzarini *et al.*, 2009). In addition, adamantane derivatives are known to exhibit marked anti­bacterial activity (Kadi *et al.*, 2010; Protopopova *et al.*, 2005). In an earlier publication, we reported the synthesis and potent anti­microbial and anti-inflammatory activities of the title compound and its related derivatives (Al-Deeb *et al.*, 2006).

### S2. Structural commentary

In the title compound (Fig. 1), the 1,2,4-triazole (N1—N3/C7/C8) ring is nearly planar with a maximum deviation of -0.007 (1) Å at atom N1. The phenyl (C1–C6) ring is almost perpendicular to the near planar 1,2,4-triazole ring with a dihedral angle of 89.61 (5) Å between them. An adamantane group is substituted at the 3-position of the triazole­thione ring. The crystal studied was an inversion twin with a 0.50 (3):0.50 (3) domain ratio.

### S3. Supra­molecular features

In the crystal packing (Fig. 2), the molecules are linked by inter­molecular N3–H1N3···S1 hydrogen bonding inter­actions forming chains extending along along the *c* axis direction. The crystal packing is further stabilized by weak C–H···π (phenyl) inter­actions (Table 1) that link the adjacent chains into a two dimensional structure in the *bc* plane.

### S4. Synthesis and crystallization

The title compound was prepared by a literature procedure (Al-Deeb *et al.*, 2006) and crystallized from EtOH/CHCl_3_ (1:1) to yield colorless crystals. M·P.: >300 °C.

^1^H NMR (CDCl_3_, 700.17 MHz): δ 1.55-1.70 (m, 6H, Adamantane-H), 1.86-2.05 (m, 9H, Adamantane-H), 7.25 (d, 2H, Ar—H, J = 8.5 Hz), 7.45 (d, 2H, Ar—H, J = 8.5 Hz), 11.90 (br. s, 1H, NH). 13C NMR (CDCl_3_, 176.08 MHz): δ 27.68, 36.03, 36.44, 39.80 (Adamantane-C), 129.95, 131.02, 134.23, 136.42 (Ar—C), 158.41 (C=N), 170.0 (C=S).

### S5. Refinement details

The nitro­gen-bound H-atom was located in a difference Fourier map and its coordinates and isotropic displacement parameter were refined freely with d(N–H) = 0.92 (2) Å. Other H atoms were positioned geometrically (d(C–H) 0.93–0.98 Å) and refined using a riding model with *U*_iso_(H) = 1.2 *U*_eq_(C). The crystal studied was an inversion twin with a 0.50 (3):0.50 (3) domain ratio.

### Crystal data

**Table:**

C_18_H_20_ClN_3_S	*D*_x_ = 1.340 Mg m^−^^3^
*M**_r_* = 345.88	Mo *K*α radiation, λ = 0.71073 Å
Tetragonal, *I*4	Cell parameters from 9155 reflections
*a* = 23.1302 (5) Å	θ = 2.5–40.8°
*c* = 6.4100 (2) Å	µ = 0.35 mm^−^^1^
*V* = 3429.39 (18) Å^3^	*T* = 150 K
*Z* = 8	Needle, colourless
*F*(000) = 1456	0.68 × 0.29 × 0.26 mm

### Data collection

**Table:**

Bruker APEXII CCD diffractometer	*R*_int_ = 0.033
φ and ω scans	θ_max_ = 41.2°, θ_min_ = 2.5°
106262 measured reflections	*h* = −42→42
11408 independent reflections	*k* = −42→42
10584 reflections with *I* > 2σ(*I*)	*l* = −11→11

### Refinement

**Table:**

Refinement on *F*^2^	Hydrogen site location: mixed
Least-squares matrix: full	H atoms treated by a mixture of independent and constrained refinement
*R*[*F*^2^ > 2σ(*F*^2^)] = 0.030	*w* = 1/[σ^2^(*F*_o_^2^) + (0.0483*P*)^2^ + 0.551*P*] where *P* = (*F*_o_^2^ + 2*F*_c_^2^)/3
*wR*(*F*^2^) = 0.082	(Δ/σ)_max_ = 0.002
*S* = 1.06	Δρ_max_ = 0.53 e Å^−^^3^
11408 reflections	Δρ_min_ = −0.31 e Å^−^^3^
213 parameters	Absolute structure: Flack (1983), 5353 Friedel pairs
0 restraints	Absolute structure parameter: 0.50 (3)

### Special details

**Table:**

Geometry. All e.s.d.'s (except the e.s.d. in the dihedral angle between two l.s. planes) are estimated using the full covariance matrix. The cell e.s.d.'s are taken into account individually in the estimation of e.s.d.'s in distances, angles and torsion angles; correlations between e.s.d.'s in cell parameters are only used when they are defined by crystal symmetry. An approximate (isotropic) treatment of cell e.s.d.'s is used for estimating e.s.d.'s involving l.s. planes.
Refinement. Refined as a 2-component inversion twin.

### Fractional atomic coordinates and isotropic or equivalent isotropic displacement parameters (Å^2^)

**Table:**

	*x*	*y*	*z*	*U*_iso_*/*U*_eq_	
S1	0.25120 (2)	0.81338 (2)	0.20570 (4)	0.01558 (4)	
Cl1	0.10286 (2)	0.98207 (2)	0.89359 (5)	0.03118 (7)	
N1	0.15119 (3)	0.77326 (3)	0.38819 (12)	0.01309 (10)	
N2	0.13872 (4)	0.68822 (4)	0.23712 (14)	0.01734 (13)	
N3	0.18691 (3)	0.71564 (4)	0.16208 (13)	0.01642 (12)	
C1	0.16658 (4)	0.83033 (4)	0.70574 (15)	0.01686 (13)	
H1A	0.1901	0.8012	0.7588	0.020*	
C2	0.15555 (5)	0.87980 (4)	0.82355 (16)	0.01990 (15)	
H2A	0.1715	0.8840	0.9558	0.024*	
C3	0.12041 (5)	0.92258 (4)	0.74046 (17)	0.01972 (15)	
C4	0.09818 (5)	0.91871 (4)	0.53962 (19)	0.02225 (17)	
H4A	0.0761	0.9486	0.4843	0.027*	
C5	0.10952 (4)	0.86925 (4)	0.42249 (16)	0.01916 (15)	
H5A	0.0953	0.8660	0.2874	0.023*	
C6	0.14220 (4)	0.82486 (4)	0.50837 (14)	0.01374 (12)	
C7	0.19637 (3)	0.76715 (4)	0.25068 (13)	0.01323 (12)	
C8	0.11738 (4)	0.72353 (4)	0.37613 (14)	0.01404 (12)	
C9	0.06417 (3)	0.70942 (4)	0.50180 (14)	0.01456 (12)	
C10	0.04492 (5)	0.64750 (5)	0.4465 (2)	0.02441 (19)	
H10A	0.0368	0.6450	0.2983	0.029*	
H10B	0.0758	0.6206	0.4784	0.029*	
C11	−0.00943 (6)	0.63104 (5)	0.5707 (2)	0.0292 (2)	
H11A	−0.0214	0.5918	0.5330	0.035*	
C12	−0.05832 (5)	0.67338 (6)	0.5199 (2)	0.0292 (2)	
H12A	−0.0673	0.6716	0.3722	0.035*	
H12B	−0.0928	0.6628	0.5971	0.035*	
C13	−0.03997 (4)	0.73459 (6)	0.5782 (2)	0.02497 (19)	
H13A	−0.0715	0.7615	0.5465	0.030*	
C14	−0.02609 (5)	0.73732 (7)	0.8112 (2)	0.0307 (2)	
H14A	−0.0145	0.7763	0.8487	0.037*	
H14B	−0.0602	0.7274	0.8916	0.037*	
C15	0.02267 (5)	0.69501 (7)	0.86116 (18)	0.0303 (3)	
H15A	0.0313	0.6967	1.0107	0.036*	
C16	0.07706 (4)	0.71160 (6)	0.73728 (16)	0.0253 (2)	
H16A	0.1082	0.6850	0.7703	0.030*	
H16B	0.0893	0.7503	0.7759	0.030*	
C17	0.00415 (6)	0.63344 (7)	0.8040 (3)	0.0356 (3)	
H17A	0.0349	0.6065	0.8369	0.043*	
H17B	−0.0298	0.6226	0.8838	0.043*	
C18	0.01416 (4)	0.75172 (5)	0.45273 (18)	0.02095 (16)	
H18A	0.0256	0.7908	0.4888	0.025*	
H18B	0.0055	0.7507	0.3047	0.025*	
H1N3	0.2084 (9)	0.6995 (9)	0.057 (4)	0.029 (5)*	

### Atomic displacement parameters (Å^2^)

**Table:**

	*U*^11^	*U*^22^	*U*^33^	*U*^12^	*U*^13^	*U*^23^
S1	0.01413 (8)	0.01610 (8)	0.01653 (8)	−0.00195 (6)	0.00022 (6)	0.00131 (6)
Cl1	0.04393 (16)	0.01649 (9)	0.03312 (14)	0.00301 (9)	0.01278 (12)	−0.00681 (9)
N1	0.0119 (2)	0.0135 (2)	0.0139 (3)	0.00076 (19)	0.0009 (2)	−0.0017 (2)
N2	0.0145 (3)	0.0176 (3)	0.0200 (3)	−0.0026 (2)	0.0042 (2)	−0.0047 (2)
N3	0.0140 (3)	0.0175 (3)	0.0178 (3)	−0.0019 (2)	0.0040 (2)	−0.0052 (2)
C1	0.0189 (3)	0.0158 (3)	0.0158 (3)	0.0034 (2)	−0.0032 (3)	−0.0026 (3)
C2	0.0251 (4)	0.0178 (3)	0.0168 (4)	0.0013 (3)	−0.0005 (3)	−0.0038 (3)
C3	0.0226 (4)	0.0136 (3)	0.0230 (4)	0.0011 (3)	0.0052 (3)	−0.0030 (3)
C4	0.0248 (4)	0.0151 (3)	0.0269 (5)	0.0065 (3)	−0.0008 (3)	0.0000 (3)
C5	0.0211 (4)	0.0170 (3)	0.0193 (4)	0.0048 (3)	−0.0030 (3)	0.0002 (3)
C6	0.0133 (3)	0.0127 (3)	0.0152 (3)	0.0021 (2)	0.0000 (2)	−0.0015 (2)
C7	0.0117 (3)	0.0146 (3)	0.0134 (3)	0.0007 (2)	0.0000 (2)	−0.0005 (2)
C8	0.0120 (3)	0.0152 (3)	0.0149 (3)	0.0001 (2)	0.0010 (2)	−0.0016 (2)
C9	0.0113 (3)	0.0177 (3)	0.0146 (3)	−0.0002 (2)	0.0011 (2)	0.0002 (3)
C10	0.0227 (4)	0.0184 (4)	0.0322 (5)	−0.0046 (3)	0.0090 (4)	−0.0016 (3)
C11	0.0251 (5)	0.0242 (4)	0.0382 (6)	−0.0082 (4)	0.0103 (4)	0.0016 (4)
C12	0.0150 (4)	0.0438 (6)	0.0288 (5)	−0.0089 (4)	−0.0016 (3)	0.0016 (5)
C13	0.0125 (3)	0.0319 (5)	0.0305 (5)	0.0038 (3)	0.0031 (3)	0.0058 (4)
C14	0.0208 (4)	0.0435 (7)	0.0280 (5)	−0.0009 (4)	0.0101 (4)	−0.0069 (5)
C15	0.0181 (4)	0.0568 (8)	0.0161 (4)	−0.0046 (4)	0.0023 (3)	0.0064 (4)
C16	0.0140 (3)	0.0462 (6)	0.0157 (4)	−0.0035 (4)	−0.0010 (3)	0.0036 (4)
C17	0.0241 (5)	0.0438 (7)	0.0388 (7)	0.0009 (5)	0.0085 (5)	0.0235 (6)
C18	0.0134 (3)	0.0238 (4)	0.0256 (4)	0.0034 (3)	0.0018 (3)	0.0055 (3)

### Geometric parameters (Å, º)

**Table:**

S1—C7	1.6836 (9)	C10—H10A	0.9700
Cl1—C3	1.7384 (10)	C10—H10B	0.9700
N1—C7	1.3744 (11)	C11—C17	1.529 (2)
N1—C8	1.3931 (11)	C11—C12	1.531 (2)
N1—C6	1.4355 (11)	C11—H11A	0.9800
N2—C8	1.3056 (12)	C12—C13	1.5246 (19)
N2—N3	1.3696 (11)	C12—H12A	0.9700
N3—C7	1.3379 (12)	C12—H12B	0.9700
N3—H1N3	0.92 (2)	C13—C14	1.529 (2)
C1—C6	1.3909 (13)	C13—C18	1.5399 (15)
C1—C2	1.3946 (13)	C13—H13A	0.9800
C1—H1A	0.9300	C14—C15	1.527 (2)
C2—C3	1.3869 (14)	C14—H14A	0.9700
C2—H2A	0.9300	C14—H14B	0.9700
C3—C4	1.3892 (16)	C15—C17	1.532 (2)
C4—C5	1.3933 (14)	C15—C16	1.5362 (15)
C4—H4A	0.9300	C15—H15A	0.9800
C5—C6	1.3889 (12)	C16—H16A	0.9700
C5—H5A	0.9300	C16—H16B	0.9700
C8—C9	1.5067 (12)	C17—H17A	0.9700
C9—C16	1.5394 (14)	C17—H17B	0.9700
C9—C10	1.5413 (14)	C18—H18A	0.9700
C9—C18	1.5473 (13)	C18—H18B	0.9700
C10—C11	1.5359 (15)		
			
C7—N1—C8	107.83 (7)	C17—C11—H11A	109.4
C7—N1—C6	122.67 (7)	C12—C11—H11A	109.4
C8—N1—C6	129.42 (7)	C10—C11—H11A	109.4
C8—N2—N3	104.93 (7)	C13—C12—C11	109.64 (9)
C7—N3—N2	113.35 (7)	C13—C12—H12A	109.7
C7—N3—H1N3	125.9 (14)	C11—C12—H12A	109.7
N2—N3—H1N3	120.7 (14)	C13—C12—H12B	109.7
C6—C1—C2	119.54 (8)	C11—C12—H12B	109.7
C6—C1—H1A	120.2	H12A—C12—H12B	108.2
C2—C1—H1A	120.2	C12—C13—C14	109.65 (11)
C3—C2—C1	118.99 (9)	C12—C13—C18	109.72 (10)
C3—C2—H2A	120.5	C14—C13—C18	109.20 (9)
C1—C2—H2A	120.5	C12—C13—H13A	109.4
C2—C3—C4	121.80 (9)	C14—C13—H13A	109.4
C2—C3—Cl1	119.00 (8)	C18—C13—H13A	109.4
C4—C3—Cl1	119.20 (8)	C15—C14—C13	109.46 (10)
C3—C4—C5	118.85 (9)	C15—C14—H14A	109.8
C3—C4—H4A	120.6	C13—C14—H14A	109.8
C5—C4—H4A	120.6	C15—C14—H14B	109.8
C6—C5—C4	119.73 (9)	C13—C14—H14B	109.8
C6—C5—H5A	120.1	H14A—C14—H14B	108.2
C4—C5—H5A	120.1	C14—C15—C17	109.82 (10)
C5—C6—C1	120.93 (8)	C14—C15—C16	109.66 (11)
C5—C6—N1	118.74 (8)	C17—C15—C16	109.74 (11)
C1—C6—N1	120.33 (7)	C14—C15—H15A	109.2
N3—C7—N1	103.86 (7)	C17—C15—H15A	109.2
N3—C7—S1	128.03 (7)	C16—C15—H15A	109.2
N1—C7—S1	128.11 (7)	C15—C16—C9	109.88 (8)
N2—C8—N1	110.01 (8)	C15—C16—H16A	109.7
N2—C8—C9	122.57 (8)	C9—C16—H16A	109.7
N1—C8—C9	127.42 (8)	C15—C16—H16B	109.7
C8—C9—C16	111.04 (7)	C9—C16—H16B	109.7
C8—C9—C10	108.32 (7)	H16A—C16—H16B	108.2
C16—C9—C10	108.17 (9)	C11—C17—C15	108.97 (10)
C8—C9—C18	111.41 (8)	C11—C17—H17A	109.9
C16—C9—C18	108.87 (8)	C15—C17—H17A	109.9
C10—C9—C18	108.96 (8)	C11—C17—H17B	109.9
C11—C10—C9	110.34 (9)	C15—C17—H17B	109.9
C11—C10—H10A	109.6	H17A—C17—H17B	108.3
C9—C10—H10A	109.6	C13—C18—C9	109.81 (8)
C11—C10—H10B	109.6	C13—C18—H18A	109.7
C9—C10—H10B	109.6	C9—C18—H18A	109.7
H10A—C10—H10B	108.1	C13—C18—H18B	109.7
C17—C11—C12	109.66 (11)	C9—C18—H18B	109.7
C17—C11—C10	109.25 (11)	H18A—C18—H18B	108.2
C12—C11—C10	109.63 (10)		
			
C8—N2—N3—C7	0.12 (11)	N1—C8—C9—C10	−175.22 (9)
C6—C1—C2—C3	−0.08 (15)	N2—C8—C9—C18	−115.92 (10)
C1—C2—C3—C4	−3.09 (16)	N1—C8—C9—C18	64.95 (12)
C1—C2—C3—Cl1	175.90 (8)	C8—C9—C10—C11	−179.89 (10)
C2—C3—C4—C5	2.89 (17)	C16—C9—C10—C11	59.67 (12)
Cl1—C3—C4—C5	−176.10 (9)	C18—C9—C10—C11	−58.54 (13)
C3—C4—C5—C6	0.48 (16)	C9—C10—C11—C17	−60.75 (14)
C4—C5—C6—C1	−3.62 (15)	C9—C10—C11—C12	59.44 (14)
C4—C5—C6—N1	177.30 (9)	C17—C11—C12—C13	59.98 (13)
C2—C1—C6—C5	3.41 (14)	C10—C11—C12—C13	−59.96 (14)
C2—C1—C6—N1	−177.52 (9)	C11—C12—C13—C14	−59.57 (12)
C7—N1—C6—C5	88.15 (11)	C11—C12—C13—C18	60.37 (13)
C8—N1—C6—C5	−88.12 (12)	C12—C13—C14—C15	59.55 (12)
C7—N1—C6—C1	−90.93 (11)	C18—C13—C14—C15	−60.70 (14)
C8—N1—C6—C1	92.80 (11)	C13—C14—C15—C17	−59.99 (13)
N2—N3—C7—N1	−0.81 (10)	C13—C14—C15—C16	60.67 (14)
N2—N3—C7—S1	178.68 (7)	C14—C15—C16—C9	−60.06 (15)
C8—N1—C7—N3	1.15 (9)	C17—C15—C16—C9	60.65 (14)
C6—N1—C7—N3	−175.82 (8)	C8—C9—C16—C15	−178.05 (10)
C8—N1—C7—S1	−178.34 (7)	C10—C9—C16—C15	−59.33 (13)
C6—N1—C7—S1	4.69 (12)	C18—C9—C16—C15	58.94 (13)
N3—N2—C8—N1	0.64 (10)	C12—C11—C17—C15	−59.90 (12)
N3—N2—C8—C9	−178.62 (8)	C10—C11—C17—C15	60.27 (13)
C7—N1—C8—N2	−1.17 (10)	C14—C15—C17—C11	60.10 (13)
C6—N1—C8—N2	175.53 (9)	C16—C15—C17—C11	−60.52 (13)
C7—N1—C8—C9	178.05 (8)	C12—C13—C18—C9	−59.95 (12)
C6—N1—C8—C9	−5.25 (15)	C14—C13—C18—C9	60.26 (13)
N2—C8—C9—C16	122.54 (11)	C8—C9—C18—C13	178.01 (9)
N1—C8—C9—C16	−56.58 (13)	C16—C9—C18—C13	−59.20 (12)
N2—C8—C9—C10	3.91 (13)	C10—C9—C18—C13	58.56 (12)

### Hydrogen-bond geometry (Å, º)

Cg2 is the centroid of the C1–C6 phenyl ring.

**Table:**

*D*—H···*A*	*D*—H	H···*A*	*D*···*A*	*D*—H···*A*
N3—H1*N*3···S1^i^	0.92 (2)	2.46 (2)	3.3253 (9)	158.4 (19)
C13—H13*A*···*Cg*2^ii^	0.98	2.97	3.8881 (13)	156

Symmetry codes: (i) −*x*+1/2, −*y*+3/2, *z*−1/2; (ii) *y*−1, −*x*+1, −*z*+1.
