# Supplementary figures and images for: Crystal structure of 3-(adamantan-1-yl)-4-(4-chloro­phen­yl)-1H-1,2,4-triazole-5(4H)-thione
Source: Acta Crystallogr E Crystallogr Commun. 2015 Jan 17;71(Pt 2):o115–6. doi: 10.1107/S2056989015000596 (PMC4384613; doi:10.1107/S2056989015000596)

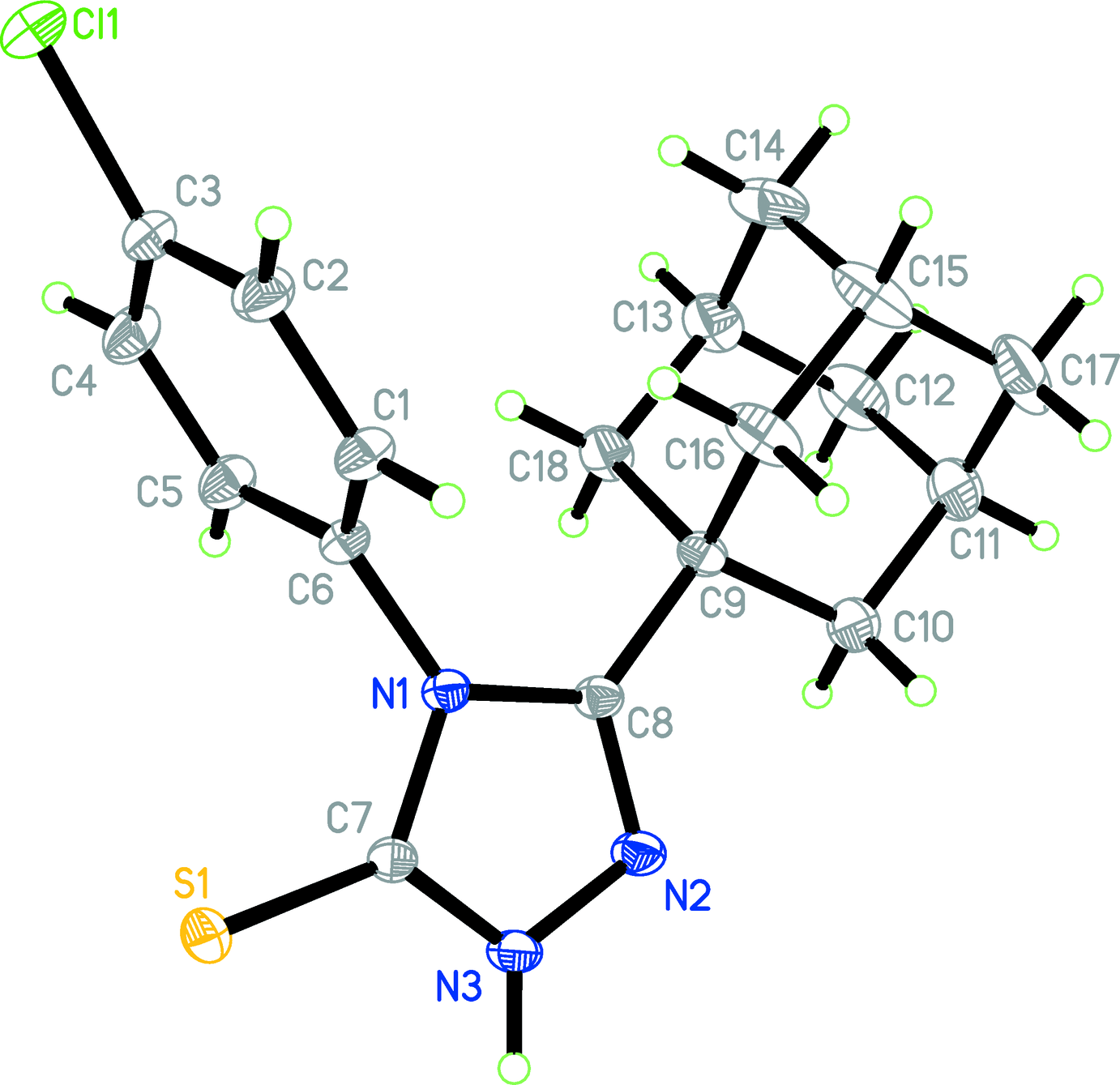

Supplement: Supplementary file 4 [file e-71-0o115-fig1.tif]

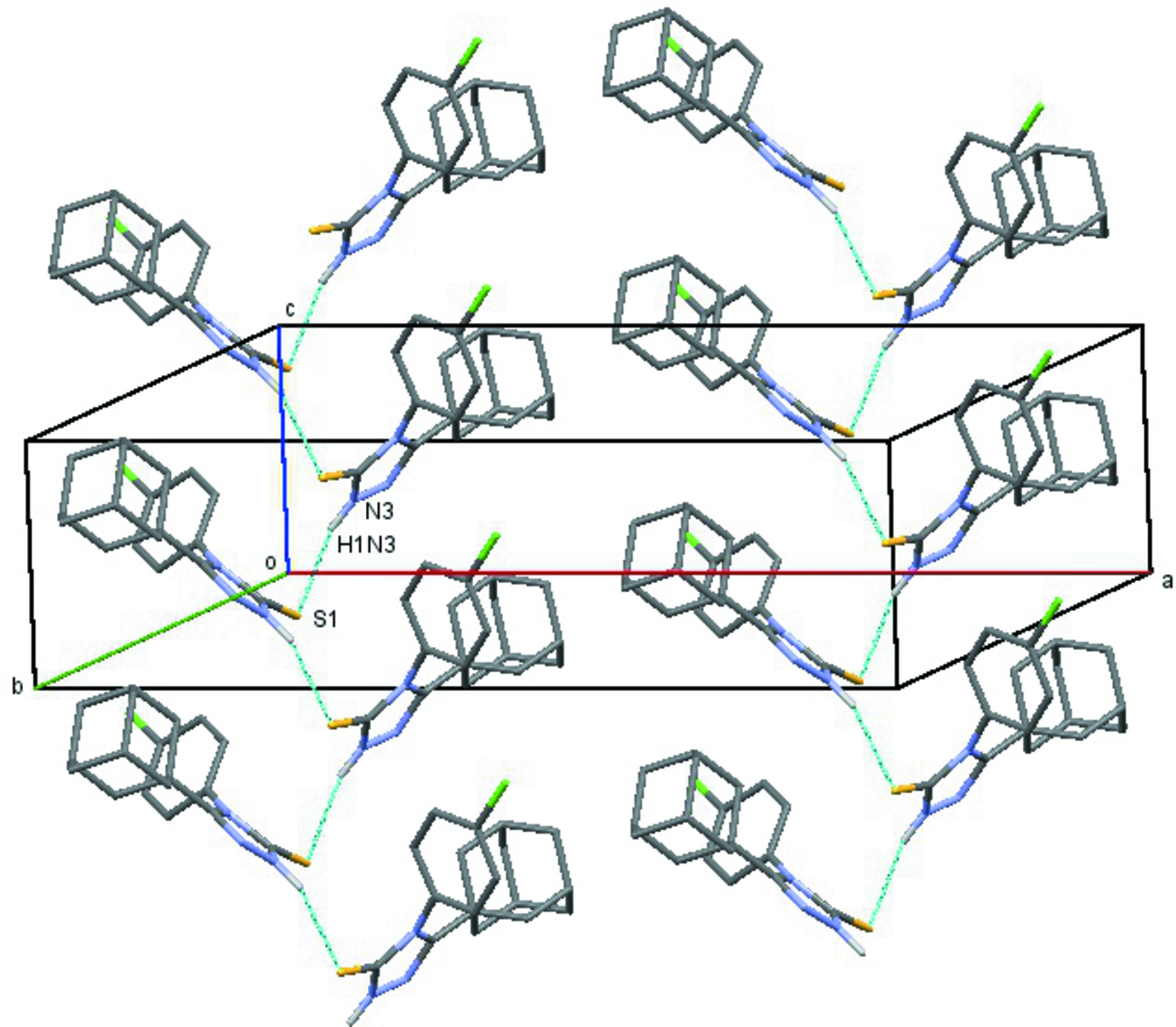

Supplement: Supplementary file 5 [file e-71-0o115-fig2.tif]
